# X-Ray Nanotomography of Individual Pulp Fibre Bonds Reveals the Effect of Wall Thickness on Contact Area

**DOI:** 10.1038/s41598-018-37380-2

**Published:** 2019-03-12

**Authors:** T. Sormunen, A. Ketola, A. Miettinen, J. Parkkonen, E. Retulainen

**Affiliations:** 1 Department of Physics, University of Jyväskylä, Jyväskylä, 40014 Finland; 2 Present Address: VTT Technical Research Centre of Finland, Oulu, 90571 Finland; 3 VTT Technical Research Centre of Finland, Jyväskylä, 40101 Finland; 4 Present Address: Swiss Light Source, Paul Scherrer Institute, Villigen, 5234 Switzerland; 5 Centre d’Imagerie BioMédicale, École polytechnique fédérale de Lausanne, Lausanne, 1015 Switzerland

**Keywords:** Biomaterials, X-rays, Imaging techniques

## Abstract

Fibre bonds play an essential role in various properties of paper. Much research has focused on their strength, but the determination of the actual contact area also provides a challenge. Many of the research methods rely on optical tools, which are restricted by the wavelength of light that is utilised. Novel X-ray computed tomography devices utilise X-rays in studying the inner structure of materials, and surpass the optical methods in terms of resolution, allowing detection of even smaller details and variations in distance between the fibres in the bond intersection area. X-ray nanotomography was used to image 26 individual cellulose fibre bonds made of springwood and summerwood fibres of refined bleached softwood kraft pulp. Various dimensional properties of the bonds were measured, most importantly the relative contact area (apparent contact area/intersection area), whose values showed wide variation from 6.4 to 85% with an average of 57.7%. Although the summerwood bonds had a somewhat smaller intersection and contact area than springwood bonds, there were no significant differences in the relative contact area between the bond types. This suggests that the effect of relative and absolute contact area on the strength differences between bond types seems to be minor.

## Introduction

Fibre bonds have been established to be an important contributor to the optical and mechanical properties of paper. The strength of fibre bonds is linked to the contact area between the fibres and is shown to have a direct effect on the strength of paper. The structure and area of individual fibre bonds are therefore of particular interest, when paper properties are to be improved. Most research on fibre contacts have utilised visible light^1–7^, and an assumption has been made that the area of optical contact is the area of *actual* molecular contact. Since the resolution of optical devices is at best 200 nm, it may not be good enough to draw a conclusion about the actual contact area.

X-ray nanotomography is a non-invasive imaging method capable of resolutions up to tens of nanometres, greatly surpassing that of optical methods. As such, it seems highly applicable in studying the contact areas of individual fibre bonds.

The difference in breaking strength and optically bonded area (OBA) between individual fibre bonds made from exclusively summerwood or exclusively springwood fibres has been recognised^4–6^, as well as bonds made of a springwood and a summerwood fibre^7^. Springwood fibre bonds have larger OBA and lower breaking strength than summerwood bonds. Spring-to-summerwood bonds have respective values between those two.

In terms of the relative contact area (RCA) of individual fibre bonds, several different studies have been conducted over the years. One of the earliest examples is the study conducted by Page *et al*.^3^ where it was found via optical microscopy that refined spruce sulphite fibre bonds had an RCA of 71.6%. In a more recent study by Kappel utilising microtome sectioning^1^, refined and unrefined softwood fibre bonds were found to have an average RCA of 98% and 86%, respectively. In the previous study utilising X-ray nanotomography^8^, the average RCA of refined softwood fibre bonds was found to be approximately 58%. An identical value was found in another study utilising confocal laser microscopy^2^. As such, it is as of yet unclear in which range the actual values lie, due to sample size and resolution differences of the aforementioned methods.

The main goal of this study was to generate a more significant data set as a continuation of the previous study^8^. In addition, another topic of interest was to find out the differences between different bond types (spring-to-springwood, spring-to-summerwood, summer-to-summerwood) in terms of contact area, relative contact area (RCA) and number of contact regions within the overlapping (intersection) area. A more detailed description of the present study can be found in a thesis by Sormunen^9^.

## Methods

### Sample preparation and imaging

The raw material used was commercially bleached kraft softwood pulp refined to 25° SR. The pulp contained mainly pine (Pinus sylvestris), but also some spruce (Picea abies) fibres may have been included. In order to facilitate the trimming of samples with a laser cutter (by increasing laser absorption), 1 litre of fibre suspension diluted to 0.01% was stained with 0.00005 M acridine orange for 10 minutes. Afterwards, the fibres were washed with purified water to get rid of excess stain. It was confirmed earlier that the staining does not weaken paper strength.

**Figure 1:**
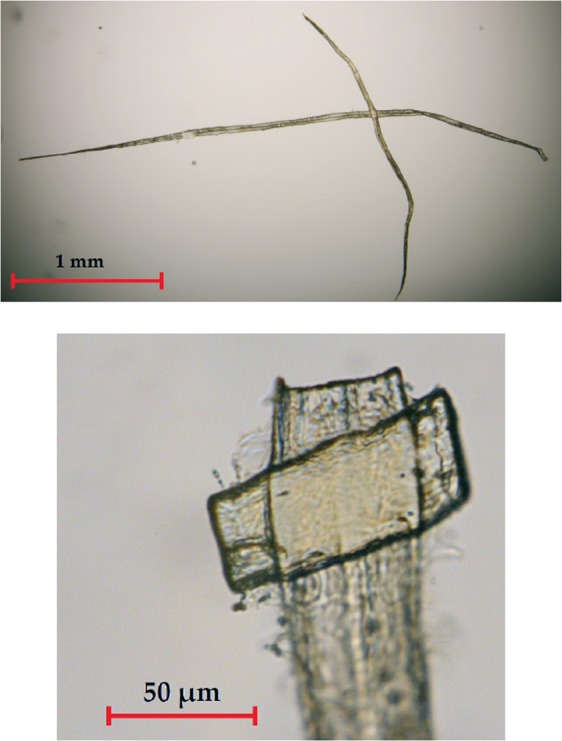
A prepared fibre bond sample (top), which was trimmed with a laser (bottom).

In order to manufacture individual fibre bonds, droplets of diluted fibre suspension were placed between two polystyrene plates and pressed with 50 kPa for 5 minutes. The plates were dried in an oven at a temperature of 80 °C for 90 minutes. Afterwards, the selected fibre bonds were glued to a needle, excess dangling ends of the fibres were trimmed (see Fig. 1) with ESI QuikLaze-50 laser cutter, and a gold marker particle was placed on top of the intersection area to facilitate image registration performed during the X-ray tomographic imaging process.

Xradia nanoXCT-100 was used for tomographic imaging. The resolution of the apparatus was empirically determined to be 128 nm (corresponding to Modulation Transfer Function value 10%), with a pixel size of 65 nm. The temperature and the relative humidity inside the machine were 25 °C and 30%, respectively. By rotating the sample 1,081 shadowgraphs were taken from various angles forming a semicircle by using 160 seconds of exposure. This meant that imaging took 48 hours per sample. Reference (flat field) correction image was obtained by averaging 40 images taken with 80 seconds of exposure time. After imaging, the shadowgraphs were aligned with the help of the marker particle by a developed automatic algorithm, and reconstruction was conducted with a filtered back-projection method.

### Image processing and analysis

Image processing and analysis were conducted using the ImageJ software^10^ and calculations were done with MATLAB. For image de-noising, Gaussian filter with radius 2 was used for the raw reconstruction stack. Furthermore, the stack was straightened with respect to the sample, and cropped accordingly. Otsu’s method^11^ was used to segment the image into binary parts: the fibre segments and the background (including spaces between fibres in the bond area). Since the contrast in the shadowgraphs was not optimal, further de-noising had to be done using a volume-opening algorithm, effectively eliminating small and isolated regions caused by imaging noise. The volume parameters 100,000 and 1,000 were used to delete white and black noise speckles, respectively.

**Figure 2:**
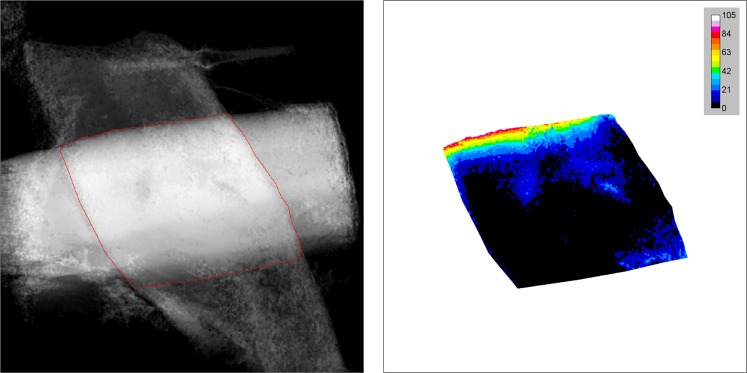
Sum projection, i.e., thickness image, with fibre intersection selected (left), and opening thickness image (right) of the same fibre bond sample (scale bar values in pixels). The image size is 65 × 65 micrometres in both cases.

A re-slicing operation was done to view the stack normally in the bond area. A sum projection image was calculated, and from this the intersection area is selected visually (see Fig. 2). The selection was used to remove the fibre segments outside the intersection area. Afterwards, the background was removed from each volume image individually, leaving only the data of the gaps between the fibres.

A similar sum projection as before was done for the modified stack. This generated the so called “opening thickness” map of the intersection area (Fig. 2), where the distance between the fibres was manifested by grey values: regions of contact had a value of 0. From this image, the relative contact area (RCA) and the cumulative separation distance distribution of intersection area could easily be calculated.

## Results

**Figure 3:**
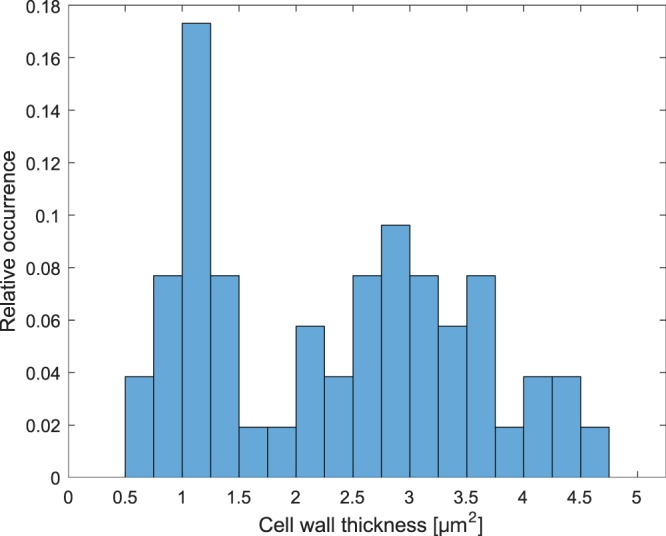
Cell wall thickness distribution of the fibres in the present study.

In total, 13 spring-to-summerwood, 7 summer-to-summerwood and 6 spring-to-springwood fibre bonds were successfully imaged. The categorisation of fibres and fibre bonds were done post-imaging with the cell wall thickness as the criterion. In this study, fibres with cell wall thickness under 2.31 μm were deemed springwood fibres and those above 2.31 μm were deemed summerwood fibres. The fibre wall thickness distribution (shown in Fig. 3) had two peaks and resembled that of pine fibres found in another study^12^. In that study, it was found that the average cell wall thickness of springwood fibres was 2.0 μm with a standard deviation of 0.31 μm.

The RCA values, i.e., the percentage of intersection area where the distance between the fibres was at most 65 nm, ranged between 6.4% and 85% in the total population, corresponding to contact area values 64 μm^2^ and 1690 μm^2^. The intersection areas ranged between 400 μm^2^ and 2,080 μm^2^. The average RCA values did not significantly vary between bond types: the total average of the population was 57.7 ± 0.8% (Table 1). This is remarkably close to the value obtained in the previous study, even if in that case only three fibre bonds made by refined fibres were successfully imaged^8^. As expected, on average the spring-to-springwood bonds had the highest absolute contact area, but the lowest value was surprisingly found in the spring-to-summerwood bonds.

**Table 1:** Dimensional properties of different bond types, with the associated sample standard deviations.

Property (average)	Spring-to-summerwood	Summer-to-summerwood	Spring-to-springwood
Intersection area [μm^2^]	1180 ± 460	1300 ± 300	1610 ± 440
RCA [%]	58.1 ± 25.0	56 ± 21	58.3 ± 27.1
Contact area [μm^2^]	690 ± 400	750 ± 330	960 ± 540
Necking ratio	1.072 ± 0.053	1.08 ± 0.08	1.07 ± 0.02
No. of contact regions	3.4	2.4	6.5

In terms of necking, springwood fibres in spring-to-springwood bonds experienced the least necking (average value 1.081), and summerwood fibres in summer-to-summerwood bonds the most (1.217). Springwood fibres necked more (1.161) than summerwood fibres (1.101) in spring-to-summerwood bonds. This suggests that summerwood fibres were able to suppress the radial shrinkage of the partner fibre more than springwood fibres. The average ratio of necking between the partner fibres did not vary significantly between bond types.

Bonds containing springwood fibres seemed to be more susceptible to lower connectedness, as evidenced by a higher number of contact regions in spring-to-springwood fibre bonds than other bond types.

**Figure 4:**
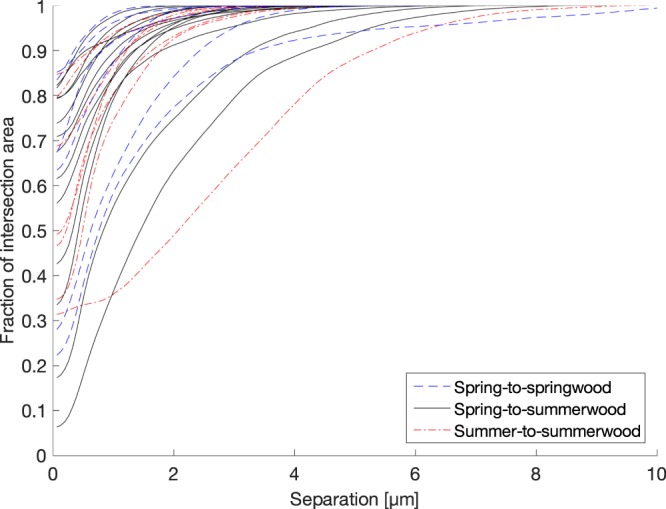
Cumulative separation distance distribution of intersection area in all fibre bond samples. The distributions are calculated from the opening thickness images.

In the case of cumulative separation distance distributions (Fig. 4), no clear differences were observed between bond types. In all cases, the distributions had the approximate shape of a sigmoid function, with distinct regions of high and low slopes. It could be speculated that unbonded areas contributing to high slopes are areas that potentially could have been bonded, but which were de-bonded after wet pressing or during drying, and low slope segments corresponding to areas that were not in contact in the first place. The wet pressing stage plays an important role by bringing together the regions of the two fibres that are morphologically compatible. After removal of the wet pressing pressure, the internal stresses may cause some spring-back in the fibre structure. The drying phase finally forms the molecular level bonds, which, however, may have broken locally due to drying stresses.

In addition to the contact area and necking measurements, correlation coefficients were calculated between relevant fibre bond properties. These are shown in Table 2. In the case of thickness, opening thickness and distance, intraspecimen correlations were calculated: the correlation coefficient was calculated between the grey values of the pixels in the opening thickness and thickness images in the intersection area, and, in the case of distance, the pixels’ distance from the centre of the intersection. Thus, each sample had thousands of data points from which the correlation was calculated. The average correlation coefficient of all samples was calculated for each bond type. In all other cases, interspecimen correlations were calculated: the correlation between RCA and other dimensional properties for each bond type was calculated. In these cases, each sample had one data point for each property.

**Table 2:** Correlation coefficients of different fibre bond properties.

Correlation between	Spring-to-summerwood	Summer-to-summerwood	Spring-to-springwood
Thickness & opening thickness	−0.481	−0.646	−0.142
Distance & opening thickness	0.406	0.391	0.363
Distance & thickness	−0.238	−0.463	0.207
Intersection area & RCA	0.085	0.178	0.259
Intersection area & contact area	0.702	0.524	0.496
Necking symmetry & RCA	0.173	0.581	0.864
No. of contact regions & RCA	−0.469	−0.278	−0.850

As can be seen, a low positive correlation was found between the distance and opening thickness of all bond types, indicating that unbonded segments are located on the edges of the intersection area. Furthermore, distance from the bond centre and bond thickness have a low negative correlation for spring-to-summerwood and summer-to-summerwood fibres, hinting that the thickness is distributed towards the centre, while in spring-to-springwood bonds, towards the edges. The negative correlation coefficient between thickness and opening thickness indicate that the greater the total thickness in a location, the greater the probability that it is in contact. This can be explained by the thicker regions experiencing a higher pressure in the wet pressing stage.

**Figure 5:**
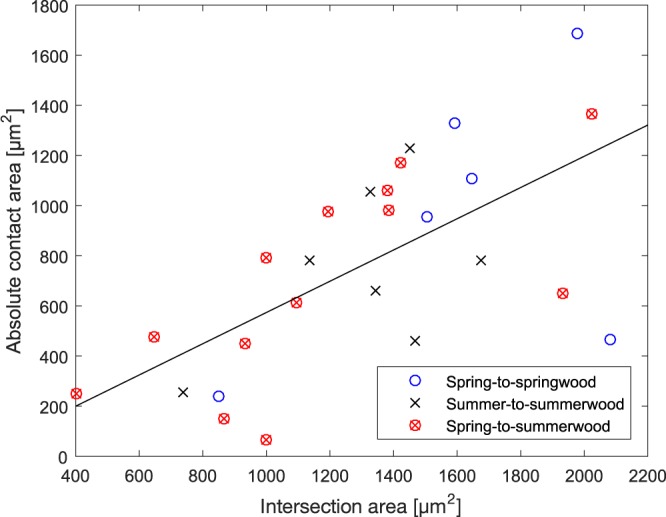
Absolute contact area vs. intersection area of imaged samples. The correlation coefficient in the whole population was found to be 0.643 (black line).

**Figure 6:**
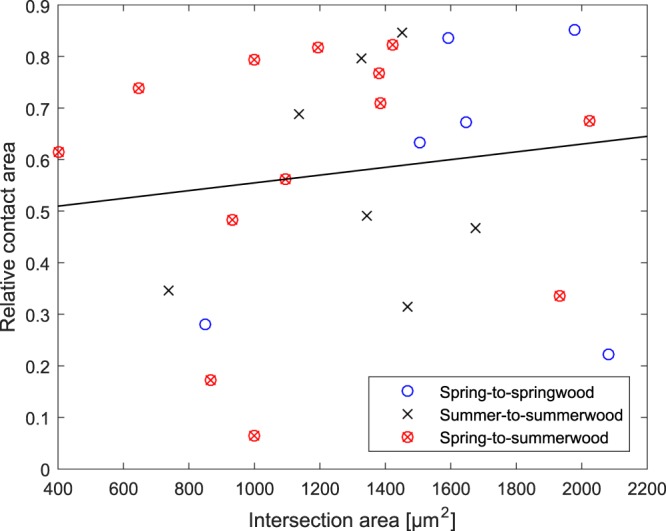
Relative contact area vs. intersection area of imaged samples. The correlation coefficient in the whole population was found to be 0.132 (black line).

Interestingly, the size of the intersection had little to no correlation with RCA (Fig. 5). The intersection area, however, was moderately correlated with the absolute contact area (Fig. 6). Necking symmetricity seemed to be connected with RCA in bonds with more symmetrical fibre combinations (summer-to-summerwood and spring-to-springwood bonds). Since necking symmetry indicates symmetric stress distribution in drying, this seems reasonable. The number of contact regions seemed to correlate negatively with RCA as well: the lower the number of contact regions the greater the RCA. This was particularly true for spring-to-springwood fibre bonds, which seemed to be more susceptible to bond region fragmentation.

## Conclusion

Continuing the previous study, a greater number of bonds made from springwood and summerwood fibres were successfully imaged. The present results suggest that in spite of the larger average contact area between springwood fibres, there seems to be no differences in spring-to-springwood, spring-to-summerwood and summer-to-summerwood fibre bonds in terms of relative contact area. However, since the number of samples is still rather low, no exact statistical conclusions can be drawn.

Several dimensional properties and correlations between them were analysed. The present data suggest that thicker summerwood fibre bonds experience a higher wet pressing pressure, which results in, e.g., high pixelwise correlation between bond thickness and the contact probability within the bond. The total fibre mass, i.e., the summed thickness of the two fibres, seems to accumulate towards the bond centre for summerwood fibres and towards the edges for springwood fibres.

The relative contact area and intersection area were found not to be correlated. Conversely, the intersection area and the absolute contact area were in moderate correlation. These findings seem to indicate that the probability of contact development is linked with the size of the overlap between the two fibres. In addition, the experienced pressure during wet pressing seems to increase the probability further. This could explain the fact that no significant differences were found in RCA between the different bond types, regardless of the established differences in intersection area between bonds made from summerwood and springwood fibres. The aforementioned points are nonetheless speculative, and a greater sample population is needed for verification.

As a tool, the X-ray nanotomography reconstruction data was found to be useful as a benchmark for other methods, but the tomographic methods need to be improved in order to generate a statistically significant data set within a more reasonable time frame. Challenges during tomographic imaging were the high failure percentage of samples (over 50% of the samples were discarded due to motion artefacts) and long imaging and sample preparation time (over 50 hours in total).

## Data Availability

The datasets generated during and/or analysed during the current study are available in a Zenodo repository^13^. Algorithms and codes are also included.
