# Publisher Correction: X-Ray Nanotomography of Individual Pulp Fibre Bonds Reveals the Effect of Wall Thickness on Contact Area

**DOI:** 10.1038/s41598-020-60473-w

**Published:** 2020-02-21

**Authors:** T. Sormunen, A. Ketola, A. Miettinen, J. Parkkonen, E. Retulainen

**Affiliations:** 1 Department of Physics, University of Jyväskylä, Jyväskylä, 40014 Finland; 2 Present Address: Vtt Technical Research Centre of Finland, Oulu, 90571 Finland; 3 VTT Technical Research Centre of Finland, Jyväskylä, 40101 Finland; 4 Present Address: Swiss Light Source, Paul Scherrer Institute, Villigen, 5234 Switzerland; 5 Centre d’Imagerie BioMédicale, École Polytechnique fédérale de Lausanne, Lausanne, 1015 Switzerland

Correction to: *Scientific Reports* 10.1038/s41598-018-37380-2, published online 12 March 2019

In the original version of this Article, the ORCID ID for Tuomas Sormunen was omitted. This has now been corrected in the HTML and PDF versions of the Article.

